# Dr. A. P. Southwick

**Published:** 1898-07

**Authors:** 


Dr. A. P. Southwick, of Buffalo, one of our best known dentists died June 5, 1898. Dr. Southwick was one of the foremost men in the dental faculty of the University of Buffalo and was largely instrumental in bringing about a substitution of electricity for the rope in capital punishment in this state. His funeral was largely attended by dentists and citizens. His burial was at Forest Lawn.

